# Economic, cultural and social inequalities in potentially inappropriate medication—An Austrian perspective

**DOI:** 10.1007/s00508-025-02638-z

**Published:** 2025-10-20

**Authors:** Christian Schoergenhofer, Thorsten Bischof, Markus Zeitlinger

**Affiliations:** Department of Clinical Pharmacology, Medical University of Vienna, Währinger Gürtel 18–20, 1090 Vienna, Austria

**Keywords:** Polypharmacy, Low socioeconomic status, Healthcare disparities, Healthcare costs, Over-treatment

## Abstract

Inappropriate prescribing remains a global challenge with implications for patient outcomes and healthcare costs. A recent Danish nationwide study investigated potentially inappropriate medications (PIMs), defined using a modified screening tool of older persons’ prescriptions (STOPP)/screening tool to alert to right treatment (START) framework and their associations with socioeconomic and sociocultural factors. The STOPP-defined PIMs, indicating potential overtreatment, were present in 3.1% and strongly associated with lower income, education, limited social support and living alone, while START-defined PIMs (12.5%), reflecting potential undertreatment, showed no such associations. These findings highlight the role of socioeconomic and cultural determinants for obtaining optimal care, even within a universal healthcare system, which minimizes cost-related barriers.

This article provides an Austrian perspective on this important topic. Available Austrian (and international) data confirm socioeconomic status as a key driver of polypharmacy and PIM exposure, although patterns vary across regions and populations. Broader evidence links PIMs to higher costs, reduced quality of life and increased hospitalizations. Addressing this complex issue requires multifaceted strategies: strengthening health literacy, educating healthcare professionals and fostering multidisciplinary collaboration, integrating deprescribing into clinical care and targeting vulnerable groups such as those with low socioeconomic status or migration backgrounds. Effective implementation of these measures may improve equity, safety and sustainability in pharmacotherapy.

In a recent Danish nationwide survey and register-based study including 177,495 patients, Paust et al. reported that 14.7% received at least 1 potentially inappropriate medication (PIM) as defined by a modified version of the screening tool of older persons’ prescriptions (STOPP) and the screening tool to alert to right treatment (START) criteria [1, 2]. The analysis primarily examined associations between PIM exposure and various social, cultural and economic factors. The findings were consistent with previous research [3, 4]: PIM use was associated with lower income and wealth, lower educational attainment, limited social support, weak social networks and living without other adults. Interestingly, although only 3.1% of the participants had at least 1 PIM defined by STOPP criteria, they were the main drivers of these associations. The STOPP criteria are often considered indicators of overtreatment. In contrast, 12.5% of the population had at least 1 PIM defined by START criteria suggestive of potential undertreatment; however, START-PIM did not show any associations with the aforementioned sociodemographic factors.

The authors made a commendable effort to include indicators of individual social capital, such as social support, cohabitation status and social network, alongside traditional risk factors like education, income and immigrant status. The observed associations remained significant after adjusting for age and sex and were consistent across both men and women. Low socioeconomic status is a well-established risk factor for chronic diseases, as also demonstrated in the Austrian Health Interview Survey (ATHIS) 2006/2007 [5]. Consequently, polypharmacy is more common and the risk of PIM is higher in patients with lower socioeconomic status [6]. On a global scale PIM use was most frequent in Africa and South America, while it occurred less frequent in Asia, Europe, North America and Oceania [7]; however, regional differences and specifics of societies must be kept in mind, e.g., concerning healthcare access and one must exercise caution when generalizing such associations. For instance, in a study conducted in Saudi Arabia, polypharmacy was more common among people with higher income, in contrast to many other publications [8]. Paust et al. investigated whether the observed associations were mediated by long-term chronic conditions and adjusted for these in their statistical models. While comorbidities partially explained the associations, most remained statistically significant after adjustment. The adjusted model represents the study’s most novel and insightful contribution and merits closer attention.

The Danish healthcare system, similar to those in Austria and other European countries, is universally accessible and free at the point of care. Medication costs exceeding 590 Euros per year are fully reimbursed. While this could theoretically explain a higher prevalence of START-defined PIMs among patients with low income, no such association was observed. In contrast, there was an almost linear association between income quintiles and STOPP-defined PIMs. This suggests that the observed associations cannot be attributed to an inability to pay for necessary medications but are more likely driven by sociocultural and educational inequalities that influence healthcare utilization.

Austrian data on this topic are primarily derived from the ATHIS. Based on ATHIS 2006/2007 data, Burkert et al. demonstrated a clear association between low socioeconomic status and unfavorable health behavior, a higher prevalence of chronic diseases, more frequent use of healthcare services and lower participation in preventive medical care [5]. A key issue in this context is how socioeconomic status is defined. Although definitions exist, such as those from the German Robert Koch Institute [9] and the European Union [10], there is no universally accepted standard, making cross-study comparisons difficult. Burkert et al. calculated the socioeconomic status using a composite index based on net income, educational level, job characteristics and self-perceived quality of life [5]. In contrast, Paust et al. employed a much more detailed approach to capture social and cultural dimensions [1]. In another Austrian study, Mayer et al. found that individuals with higher education and income were more likely to use non-prescribed medication, whereas those with a lower socioeconomic status were more likely to take prescribed drugs and consult general practitioners beforehand, highlighting differing patterns of healthcare utilization [11]. These findings align with previous research showing associations between educational attainment and exposure to PIM [12, 13]. In these studies, education is often used as a proxy for health literacy as it is easily measurable and closely linked to both health literacy and broader health outcomes [14–17]; however, this approach has limitations. For example, studies have not consistently demonstrated associations between standard health literacy scores and outcomes, such as PIM use or polypharmacy [18, 19]. It is worth noting that this relationship remains underexplored and available data are limited. Regarding health literacy itself, recent evidence paints a concerning picture for Austria: several studies rank the country among those with the lowest levels of health literacy in Europe [20, 21].

Migrants represent a highly heterogeneous segment of the population but often face a higher risk of lower socioeconomic status. A scoping review by Lebano et al. highlighted persistent barriers to healthcare access in Europe, including legal, economic, cultural and linguistic challenges as well as discrimination, despite efforts to promote equity [22]. Austrian Health Interview Survey (ATHIS) data from 2014 and 2019 showed lower healthcare utilization among individuals with a migration background, reflected by reduced vaccination rates, screening participation and dental visits [23]. Although individuals with a migration background make up approximately 24% of Austria’s population, they account for only 19.4% of total healthcare expenditure [23]. A large Austrian study of 13 million hospitalizations confirmed lower hospitalization rates among migrants, except for German migrants, whose rates resembled those of the Austrian population [24]. Readmission rates varied by country of origin: higher among individuals from Russia, Serbia or Turkey (males) and lower for those from Hungary, Romania or Turkey (females). These patterns may reflect the “healthy migrant effect”, which refers to the selective migration of healthier individuals [25], or the “salmon effect”, describing a tendency of older and/or morbid migrants to return to their countries of origin [26]. Of note, the real impact of these effects is still contradictory but they exemplify methodological challenges in such studies. In the Danish study by Paust et al., no significant association was found between migration status and PIM exposure [1]. Yet the sample of immigrants (*n* = 1406 with 173 PIMs) was small compared to the native population (*n* = 164,079, with 37,855 PIMs), and the study was not designed to assess differences between migrant and non-migrant populations.

The STOPP/START criteria are designed to improve pharmacotherapy in older adults by identifying PIMs and recommending evidence-based prescribing practices. These criteria are regularly revised by an international panel of European experts, with the most recent version (3.0), comprising 190 criteria, published in 2023 [2]. While the criteria serve as a valuable tool for research involving large populations and offer general guidance for clinical decision-making, it is important to emphasize that individual treatment decisions must always be tailored to the specific clinical context. Deviations from guideline-based criteria may be clinically appropriate in certain cases, although such individualized decisions are not captured in large-scale database studies. In the present study, a modified version of STOPP/START criteria was employed to enable their application not only to older adults but to the general adult population (> 18 years) and to ensure compatibility with the structure of Danish electronic health records [1]. Consequently, direct cross-study comparisons of PIM prevalence should be interpreted with caution, particularly when different age groups, coding systems, or adaptions of the criteria are involved. Although no Austrian population-based data are available on PIM prevalence using the original STOPP/START criteria, several cross-sectional studies in specific subpopulations indicate that PIM use remains common, e.g. in older adults with chronic renal insufficiency [27], in nursing home residents [28] and in the general older population [29]. Similar criteria have been developed or adapted to be better applicable in Austria, including the Austrian PIM list proposed by Mann et al. in 2012, the German PRISCUS 2.0 list (published in 2023 inclusive of Austrian data) [35], and the EU(7)-PIM list introduced in 2015 by a consortium of 7 European countries [36], albeit, without Austrian participation. Despite the growing availability of such lists and increasing evidence on the prevalence and risks associated with inappropriate prescribing, implementation into routine clinical practice remains limited. A qualitative study conducted in Germany in 2017 found that the majority of general practitioners were unfamiliar with the PRISCUS list [30]. Among those who were aware of it, many expressed scepticism regarding its utility and applicability in everyday clinical settings.

Several large-scale analyses have demonstrated that exposure to PIM is associated with increased healthcare costs [31–33] and reduced quality of life [33]. Notably, Alcusky et al. reported that interventions aimed at reducing PIM exposure were linked to a decreased incidence of unplanned hospitalizations [34]. These findings are particularly relevant given the demographic shifts in western societies and growing financial pressures on healthcare systems. In light of this, a multifaceted approach may represent a meaningful long-term investment to enhance both the quality and cost-efficiency of healthcare. Key strategies could include: (i) improving health literacy at the population level (potentially through integration into compulsory education), (ii) educating healthcare professionals and increasing awareness regarding appropriate pharmacotherapy, (iii) optimizing drug therapy, ideally through multidisciplinary collaboration, (iv) better implementation of deprescribing into clinical routine and (iv) reducing structural and systemic barriers to healthcare access, with a particular focus on individuals with low socioeconomic status as well as those with a migration or refugee background. Collectively, such measures could contribute to more equitable care delivery and better health outcomes while alleviating the economic burden on healthcare systems.
